# Supplementary material for: A Catalog of GNI-A1 Genes That Regulate Floret Fertility in a Diverse Bread Wheat Collection
Source: Plants (Basel). 2024 Jan 23;13(3):330. doi: 10.3390/plants13030330 (PMC10857310; doi:10.3390/plants13030330)
Supplement: Supplementary file 1 [file plants-13-00330-s001.zip › plants-2791407-supplementary.pdf]

Supplementary Table S1. Members of the NBRP bread wheat panel.

NBRP no.	Line	Species	Country	*GNI-A1* allele[1]
6x-001	KU-166	*T. aestivum*	China	Y
6x-002	KU-265 (Norin 10)	*T. aestivum*	Japan	Y
6x-003	KU-309 (Baart)	*T. aestivum*	USA	-
6x-004	KU-333 (Selkerk)	*T. aestivum*	Canada	Y
6x-005	KU-336 (Wichita)	*T. aestivum*	USA	N
6x-006	KU-366 (April Bearded)	*T. aestivum*	UK	N
6x-007	KU-370 (Renown)	*T. aestivum*	UK	N
6x-008	KU-371 (Wilhelmina)	*T. aestivum*	UK	N
6x-009	KU-372 (Little Joss)	*T. aestivum*	UK	N
6x-010	KU-373 (Victor)	*T. aestivum*	UK	N
6x-011	KU-374 (Yeoman)	*T. aestivum*	UK	N
6x-012	KU-405	*T. aestivum*	Uzbekistan	Y
6x-013	KU-479	*T. aestivum*	China	Y
6x-014	KU-481	*T. aestivum*	China	Y
6x-015	KU-483	*T. aestivum*	Tanzania	Y
6x-016	KU-497	*T. aestivum*	India	Y
6x-017	KU-504	*T. aestivum*	China	Y
6x-018	KU-601 (Gifu Wase Komugi)	*T. aestivum*	Japan	Y
6x-019	KU-1002	*T. aestivum*	Spain	Y
6x-020	KU-1005	*T. aestivum*	Spain	Y
6x-021	KU-1011	*T. aestivum*	Spain	N
6x-022	KU-1049	*T. aestivum*	Spain	K
6x-023	KU-1143	*T. aestivum*	Spain	N
6x-024	KU-1215 (Akita Zairai)	*T. aestivum*	Japan	K
6x-025	KU-1230 (Fukoku)	*T. aestivum*	Japan	Y
6x-026	KU-1279 (Murasaki Aka)	*T. aestivum*	Japan	N
6x-027	KU-1302	*T. aestivum*	Greece	N
6x-028	KU-1347	*T. aestivum*	Greece	-
6x-029	KU-1392	*T. aestivum*	Romania	Y
6x-030	KU-1394	*T. aestivum*	Romania	N
6x-031	KU-1421	*T. aestivum*	Romania	N
6x-032	KU-1424	*T. aestivum*	Romania	Y
6x-033	KU-1521	*T. aestivum*	Azerbaijan	N
6x-034	KU-1527	*T. aestivum*	Armenia	N
6x-035	KU-1644	*T. aestivum*	Armenia	Y
6x-036	KU-1668	*T. aestivum*	Georgia	N
6x-037	KU-1697	*T. aestivum*	Georgia	N
6x-038	KU-1797	*T. aestivum*	Georgia	N
6x-039	KU-3006	*T. aestivum*	Pakistan	Y
6x-040	KU-3010	*T. aestivum*	Pakistan	Y
6x-041	KU-3037	*T. aestivum*	Pakistan	Y
6x-042	KU-3045	*T. aestivum*	Afghanistan	Y
6x-043	KU-3054	*T. aestivum*	Afghanistan	Y
6x-044	KU-3062	*T. aestivum*	Afghanistan	Y
6x-045	KU-3083	*T. aestivum*	Afghanistan	N
6x-046	KU-3089	*T. aestivum*	Afghanistan	Y
6x-047	KU-3097	*T. aestivum*	Iran	Y
6x-048	KU-3098	*T. aestivum*	Iran	Y
6x-049	KU-3121	*T. aestivum*	Iran	Y
6x-050	KU-3126	*T. aestivum*	Iran	Y

6x-051	KU-3136	*T. aestivum*	Iran	Y
6x-052	KU-3162	*T. aestivum*	Iran	Y
6x-053	KU-3184	*T. aestivum*	Iran	Y
6x-054	KU-3189	*T. aestivum*	Iran	Y
6x-055	KU-3202	*T. aestivum*	Iran	Y
6x-056	KU-3232	*T. aestivum*	Iran	Y
6x-057	KU-3236	*T. aestivum*	Iran	Y
6x-058	KU-3274	*T. aestivum*	Iran	Y
6x-059	KU-3289	*T. aestivum*	Iran	Y
6x-060	KU-3299	*T. aestivum*	Pakistan	Y
6x-061	KU-3351	*T. aestivum*	Pakistan	Y
6x-062	KU-3752	*T. aestivum*	Egypt	N
6x-063	KU-3777	*T. aestivum*	Jordan	N
6x-064	KU-3778	*T. aestivum*	Lebanon	N
6x-065	KU-3780	*T. aestivum*	Syria	N
6x-066	KU-3784	*T. aestivum*	Turkey	N
6x-067	KU-3789	*T. aestivum*	Turkey	Y
6x-068	KU-3801	*T. aestivum*	Turkey	N
6x-069	KU-3806	*T. aestivum*	Turkey	N
6x-070	KU-3818	*T. aestivum*	Turkey	Y
6x-071	KU-3834	*T. aestivum*	Turkey	Y
6x-072	KU-3851	*T. aestivum*	Turkey	Y
6x-073	KU-3857	*T. aestivum*	Turkey	K
6x-074	KU-3860	*T. aestivum*	Turkey	N
6x-075	KU-3868	*T. aestivum*	Italy	N
6x-076	KU-4703	*T. aestivum*	Nepal	Y
6x-077	KU-4714	*T. aestivum*	Nepal	Y
6x-078	KU-4734	*T. aestivum*	Nepal	Y
6x-079	KU-4759	*T. aestivum*	Nepal	Y
6x-080	KU-4769	*T. aestivum*	Nepal	Y
6x-081	KU-4783	*T. aestivum*	Nepal	N
6x-082	KU-7001	*T. aestivum*	Bhutan	Y
6x-083	KU-7041	*T. aestivum*	Bhutan	Y
6x-084	KU-7113	*T. aestivum*	Bhutan	Y
6x-085	KU-7180	*T. aestivum*	Bhutan	Y
6x-086	KU-7356	*T. aestivum*	Ethiopia	Y
6x-087	KU-7379	*T. aestivum*	Ethiopia	Y
6x-088	KU-7406	*T. aestivum*	Ethiopia	Y
6x-089	KU-7437	*T. aestivum*	Afghanistan	Y
6x-090	KU-7459	*T. aestivum*	Afghanistan	Y
6x-091	KU-7480	*T. aestivum*	Afghanistan	Y
6x-092	KU-7624	*T. aestivum*	Afghanistan	Y
6x-093	KU-7653	*T. aestivum*	Afghanistan	Y
6x-094	KU-7669	*T. aestivum*	Afghanistan	Y
6x-095	KU-9431	*T. aestivum*	Ethiopia	Y
6x-096	KU-9460	*T. aestivum*	Ethiopia	-
6x-097	KU-9797	*T. aestivum*	Ethiopia	Y
6x-098	KU-9820	*T. aestivum*	Ethiopia	Y
6x-099	KU-9867	*T. aestivum*	Ethiopia	Y
6x-100	KU-10001	*T. aestivum*	Iraq	K
6x-101	KU-10154	*T. aestivum*	Iraq	Y
6x-103	KU-10393	*T. aestivum*	Iran	K
6x-105	KU-10439	*T. aestivum*	Iran	Y
6x-106	KU-10480	*T. aestivum*	Iran	N
6x-107	KU-10510	*T. aestivum*	Iran	N

6x-108	KU-11201	*T. aestivum*	Afghanistan	Y
6x-109	KU-11214	*T. aestivum*	Afghanistan	Y
6x-110	KU-11240A	*T. aestivum*	Afghanistan	-
6x-111	KU-11351	*T. aestivum*	Romania	Y
6x-112	KU-11702	*T. aestivum*	Greece	Y
6x-113	KU-11809	*T. aestivum*	Greece	K
6x-114	KU-11829	*T. aestivum*	Greece	K
6x-115	KU-13501	*T. aestivum*	China	N
6x-116	KU-13506	*T. aestivum*	China	N
6x-117	KU-13546	*T. aestivum*	China	Y
6x-118	KU-13631	*T. aestivum*	China	Y
6x-119	KU-13662	*T. aestivum*	China	Y
6x-120	KU-13708	*T. aestivum*	China	Y
6x-121	KU-13807	*T. aestivum*	China	Y
6x-122	KU-13891	*T. aestivum*	China	Y
6x-123	KU-1020	*T. spelta*	Spain	N
6x-124	KU-1062	*T. spelta*	Spain	N
6x-125	KU-1137	*T. spelta*	Spain	N
6x-126	KU-3377	*T. spelta*	Iran	Y
6x-127	KU-3401	*T. spelta*	Germany	N
6x-128	KU-3413	*T. spelta*	Germany	N
6x-129	KU-3416	*T. spelta*	Germany	N
6x-130	KU-3417	*T. spelta*	Germany	N
6x-131	KU-3421	*T. spelta*	Germany	N
6x-132	KU-3443	*T. spelta*	Germany	N
6x-133	KU-3444	*T. spelta*	Germany	N
6x-134	KU-3445	*T. spelta*	Germany	N
6x-135	KU-152	*T. compactum*	China	K
6x-136	KU-1208	*T. compactum*	Japan	Y
6x-138	KU-3063	*T. compactum*	Afghanistan	-
6x-139	KU-3242	*T. compactum*	Iran	Y
6x-140	KU-7350	*T. compactum*	Turkey	Y
6x-141	KU-9873	*T. compactum*	Ethiopia	-
6x-142	KU-197	*T. macha*	Turkey	N
6x-143	KU-1812	*T. macha*	Georgia	N
6x-144	KU-1814	*T. macha*	Georgia	N
6x-145	KU-1817	*T. macha*	Georgia	N
6x-146	KU-161	*T. sphaerococcum*	–	Y
6x-147	KU-162-2	*T. sphaerococcum*	Pakistan	Y
6x-148	KU-3004	*T. sphaerococcum*	Pakistan	-
6x-149	KU-192	*T. vavilovii*	–	Y
6x-150	KU-3848	*T. vavilovii*	Turkey	Y
6x-151	Opata 85	*T. aestivum*	Mexico	Y
6x-152	W7984	Synthetic	–	Y
6x-153	Chinese Spring	*T. aestivum*	China	Y
6x-154	KT019-001 (var. duhamerianum)	*T. spelta*	Germany	N
6x-155	Timstein	*T. aestivum*	Australia	Y
6x-156	Hope	*T. aestivum*	USA	Y
6x-157	Cheyenne	*T. aestivum*	USA	N
6x-158	Hokkai 240	*T. aestivum*	Japan	K
6x-159	Kanto 107	*T. aestivum*	Japan	Y
6x-160	Norin 26	*T. aestivum*	Japan	Y
6x-161	Norin 61	*T. aestivum*	Japan	Y
6x-163	Chihokukomugi	*T. aestivum*	Japan	K
6x-164	Nanbukomugi	*T. aestivum*	Japan	K

6x-165	Zenkojikomugi	*T. aestivum*	Japan	Y
6x-166	Sumai 3	*T. aestivum*	China	N
6x-167	Nobeokabouzu	*T. aestivum*	Japan	Y
6x-168	Tamaizumi	*T. aestivum*	Japan	Y
6x-169	Kitanokaori	*T. aestivum*	Japan	N
6x-170	Minaminokaori	*T. aestivum*	Japan	Y
6x-171	Haruyokoi	*T. aestivum*	Japan	Y
6x-172	KS831957	*T. aestivum*	USA	N
6x-173	Hanamanten	*T. aestivum*	Japan	Y
6x-175	Kinuiroha	*T. aestivum*	Japan	Y
6x-176	U24	*T. aestivum*	China	Y
6x-177	Seikai 165	*T. aestivum*	Japan	N
6x-178	Ayahikari	*T. aestivum*	Japan	Y
6x-179	Minaminokomugi	*T. aestivum*	Japan	Y
6x-180	Chogokuwase	*T. aestivum*	Japan	N
6x-181	Akimakigata-Abukumawase	*T. aestivum*	Japan	Y
6x-182	Nishikazekomugi	*T. aestivum*	Japan	Y
6x-183	Chikugoizumi	*T. aestivum*	Japan	Y
6x-184	Iwainodaichi	*T. aestivum*	Japan	Y
6x-185	Shiroganekomugi	*T. aestivum*	Japan	Y
6x-186	Seikai 193	*T. aestivum*	Japan	Y
6x-187	Kitakamikomugi	*T. aestivum*	Japan	Y
6x-188	Nebarigoshi	*T. aestivum*	Japan	K
6x-189	Shunyou	*T. aestivum*	Japan	N
6x-190	Fujimikomugi	*T. aestivum*	Japan	Y
6x-191	Gamenya	*T. aestivum*	Australia	Y
6x-192	Akadaruma	*T. aestivum*	Japan	Y
6x-193	Bobwhite	*T. aestivum*	Mexico	N

[1] *GNI-A1* allele denotes variation at the 105th amino acid (N: asparagine, Y: tyrosine, or K: lysine). "-"indicates missing genotypes.

Supplementary Table S2. Members of the HRO bread wheat panel.

Kitami no.	Line	Species	Country	*GNI-A1* allele[1]
-	Haunsberg	*T. aestivum*	Austria	N
-	Münstertaler	*T. aestivum*	Austria	N
-	Niederndorferberg	*T. aestivum*	Austria	N
-	AC Readymade	*T. aestivum*	Canada	N
-	CDC Kestrel	*T. aestivum*	Canada	N
-	Norstar	*T. aestivum*	Canada	N
-	FHBRWBW Entry 28	*T. aestivum*	CIMMYT	N
-	Bussard	*T. aestivum*	Germany	N
-	Flair	*T. aestivum*	Germany	N
-	Pegassos	*T. aestivum*	Germany	N
-	Trend-2740	*T. aestivum*	Germany	N
04903	Akasabi Shirazu1	*T. aestivum*	Japan	K
16985	Hokushin	*T. aestivum*	Japan	K
05947	Horosirikomugi	*T. aestivum*	Japan	N
25370	Kitahonami	*T. aestivum*	Japan	Y
24141	Kitamoe	*T. aestivum*	Japan	K
27268	Kitasachiho	*T. aestivum*	Japan	K
-	OW104	*T. aestivum*	Japan	N
27814	Takikeimugi2	*T. aestivum*	Japan	K

27815	Takikeimugi3	*T. aestivum*	Japan	K
05946	Takunekomugi	*T. aestivum*	Japan	K
27269	Tsurukichi	*T. aestivum*	Japan	N
-	Yumechikara	*T. aestivum*	Japan	N
-	Valujevskaja	*T. aestivum*	Russia	N
-	Arina	*T. aestivum*	Switzerland	N
-	PI 173438	*T. aestivum*	Turkey	K
-	ABBOT	*T. aestivum*	UK	N
-	BEAVER	*T. aestivum*	UK	Y
-	Blaze	*T. aestivum*	UK	N
-	Charger	*T. aestivum*	UK	Y
-	Civic	*T. aestivum*	UK	Y
-	Consort	*T. aestivum*	UK	Y
-	Haven	*T. aestivum*	UK	Y
-	Hereward	*T. aestivum*	UK	N
-	Rendezvous	*T. aestivum*	UK	N
-	Rialto	*T. aestivum*	UK	N
-	Sniper	*T. aestivum*	UK	Y
-	Soldier	*T. aestivum*	UK	N
-	Allegiance	*T. aestivum*	USA	N
-	Betty	*T. aestivum*	USA	Y
-	Brundage	*T. aestivum*	USA	N
-	Chesapeake	*T. aestivum*	USA	N
-	Co701610	*T. aestivum*	USA	N
-	Coda	*T. aestivum*	USA	K
-	Deloris	*T. aestivum*	USA	N
-	Eltan	*T. aestivum*	USA	Y
-	IDO361	*T. aestivum*	USA	N
-	Iohardi	*T. aestivum*	USA	N
-	Jagger	*T. aestivum*	USA	Y
-	Karl92	*T. aestivum*	USA	N
-	Madsen	*T. aestivum*	USA	N
-	Palo Duro	*T. aestivum*	USA	Y
-	Rely	*T. aestivum*	USA	K
-	Satanta	*T. aestivum*	USA	K
-	Stadler	*T. aestivum*	USA	N
-	Stephens	*T. aestivum*	USA	N
-	Turkey red II	*T. aestivum*	USA	N
-	Utah-100	*T. aestivum*	USA	N
-	WA7811	*T. aestivum*	USA	N
-	WA7813	*T. aestivum*	USA	Y
-	WA7833	*T. aestivum*	USA	Y
-	WA7834	*T. aestivum*	USA	K
-	Weatherford	*T. aestivum*	USA	N
-	Sava	*T. aestivum*	Yugoslavia	N

[1] *GNI-A1* allele denotes variation at the 105th amino acid (N: asparagine, Y: tyrosine, or K: lysine).
